# An Initial Damage Model of Rock Materials under Uniaxial Compression Considering Loading Rates

**DOI:** 10.3390/ma15165589

**Published:** 2022-08-15

**Authors:** Gang Meng, Zhizhen Liu, Ping Cao, Ziyang Zhang, Zhi Fan, Hang Lin, Huijuan Deng

**Affiliations:** 1 School of Resource and Safety Engineering, Central South University, Changsha 410083, China; 2 Sinohydro Engineering Bureau 8 Co., Ltd., Changsha 410004, China

**Keywords:** loading rate, mechanical behavior, initial damage, damage variable, damage constitutive model

## Abstract

Existing rock material damage models always ignore the initial damage characteristics of rock materials, and the actual rock materials have initial damage characteristics. To consider the rock’s initial damage characteristics, a series of compression tests for yellow sandstone was carried out. First, the acoustic emission characteristics and damage model of yellow sandstone, considering the loading rates, were analyzed. Second, an initial damage model, which can better describe the initial damage characteristics of yellow sandstone materials, is presented. The research results show that the strength and elastic modulus of yellow sandstone depends on the loading rate, and increases as the loading rate increases.

## 1. Introduction

**Figure 1:**
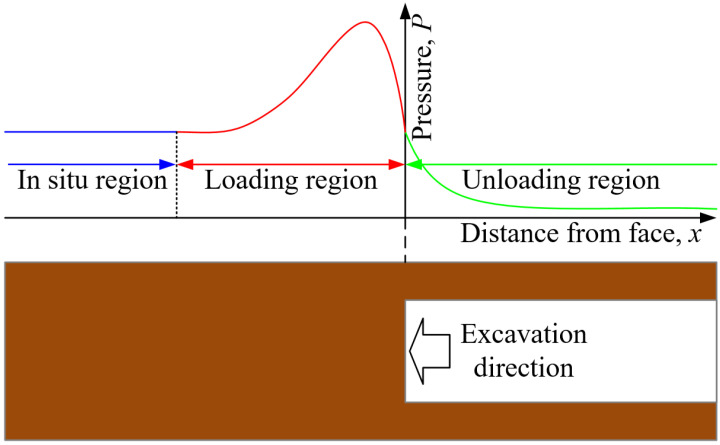
Schematic diagram of stress distribution during the excavation period [1].

The excavation of an underground tunnel will increase pressure in the surrounding rock. The excavation process is a loading process for surrounding rock that causes a significant change in the stress distributions in the loading region [1], as shown in Figure 1. It is difficult to reproduce the excavation process in the laboratory. A better method is to study the influence of excavation on the stability of surrounding rock by controlling the loading rate. This method can better reproduce the phenomenon of the increase in surrounding rock pressure caused by excavation, and reveal the nature of the influence of the increase in surrounding rock pressure on the stability of a cavern. Therefore, it is of great practical significance to study rock’s mechanical response to different loading rates.

Many scholars extensively studied the effect of loading rate on rock’s mechanical behavior. Backers et al. [2,3], Zhou et al. [4], Xing et al. [5], Lin et al. [6,7], Zhang et al. [8], and Li et al. [9] studied fracture toughness and fracture mechanics parameters subjected to different loading rates. Hashiba et al. [10] and Okubo et al. [11] studied the influence of the loading rate on peak and residual rock strengths and determined an alternating loading rate. Jeong et al. [12] studied the influence of organic steam and inorganic gas on rock’s uniaxial compressive strength under a constant loading rate. Zhao et al. [13] analyzed the activity rules of acoustic emission and electromagnetic emission under different loading rates. Komurlu et al. [14] studied the size effect of rock and rock-like materials under different displacement rates and loading rates, and found that the size effect under different loading rates was more significant than displacement rates. Feng et al. [15] studied the mechanical behaviors and energy evolution rules of intact and fractured rocks subjected to uniaxial loading considering strain loading rates. Wang et al. [16] found that the failure mode of rock samples changed from intergranular failure to transgranular failure with an increase in strain rate. Wu et al. [17] studied the influence of loading rate on dilatancy, acoustic emission, and failure characteristics of prefabricated double-crack rock. Hokka et al. [18] systematically studied the mechanical behavior of Kulu gray granite under different confining pressures and strain rates. Ma et al. [19] evaluated the comprehensive effects of strain rate on the mechanical properties of coral rocks, i.e., uniaxial compressive strength, Young’s modulus, brittleness, energy dissipation, and failure mode. Gong et al. [20] systematically studied the characteristics of single particle breakage at different strain rates. Malik et al. [21] studied the influence of loading rate on the peak stress, elastic modulus, deformation modulus, and strain energy of Deccan basalt. Mahanta et al. [22] presented critical and appropriate empirical equations to evaluate the effect of the strain rate on the mechanical properties of rock.

Acoustic emission (AE) technology is an important tool for non-destructive rock testing. Acoustic emission parameters, such as acoustic emission count, energy, and amplitude, can well reflect the propagation process of microcracks and describe the damage evolution process inside a rock [23]. Hence, AE technology is extensively used to reveal the failure mechanics of rock. Chen et al. [24] studied the AE characteristics of sandstone in the entire process of stress-strain under different loading rates. Some scholars [25,26,27,28,29] used numerical simulation software to analyze the mechanical properties and AE characteristics of rocks under different loading rates; the simulation results are in good agreement with the experimental results. Wang et al. [30] studied the failure process and AE characteristics of coal-rock composite samples under different loading rates. Cao et al. [31,32] found that with an increase in loading rate AE hits, times, and events all decreased, but AE energy increased, establishing a damage constitutive equation based on the equivalent strain hypothesis and AE. Zhao et al. [33] studied the variation rules of AE characteristics, three-dimensional position, and thermodynamic coupling damage characteristics of high-temperature granite under different loading rates.

Many AE statistical damage models study the influence of loading on the internal damage of rock material [34,35,36,37,38]. Zhang et al. [39] established an AE damage model of quasi-brittle materials and obtained the general expression of the AE rate and the Kaiser effect. Peng et al. [40] proposed an elasto-viscoplastic constitutive model of sand based on energy theory to consider the effect of stress path and loading rate. Zhao et al. [41] analyzed the damage evolution process of granite under different loading rates from the perspective of energy. The change in damage variable is accelerated with an increase in loading rate. Yang et al. [42] proposed a damage plastic constitutive model considering confining pressure and strain rate on rock strength. Qi et al. [43] proposed a new asymptotic intermediate approximation model of viscosity through the analysis of different structure levels. Saksala et al. [44] simulated the compressive behavior of granite under high strain rate dynamic loading and large confining pressure, and established a rock constitutive model based on damage mechanics.

Although many proposed damage constitutive models are used to study the microscopic damage mechanism considering different loading rates, existing damage constitutive models ignore the initial damage. To make up this deficiency in the existing research, an initial damage constitutive model is proposed. Compared with the existing damage models, such as the Weibull strength damage model and the AE damage model, the proposed initial damage model can better characterize the initial damage of rock materials. Finally, the proposed initial damage constitutive model was verified by a series of experiments.

## 2. Experimental Setup

### 2.1. Specimen Preparation

The yellow sandstone used in the experiment was collected from Sichuan Province, China. The inner structure of the rock sample was dense, and there were no obvious cracks on the surface. The rock sample was earth yellow. According to X-ray diffraction analysis, the rock sample consisted of 70.4% anorthite, 14.6% quartz, 2.8% andesite, 3.4% amphibolite, 3.2% pyroxene, and 5.6% other components. To ensure the uniformity of the test results and reduce the differences, the rock samples were all taken from the same complete yellow sand rock block. To reduce the end effect in the test, the rock was processed into a 40 mm × 40 mm × 120 mm rectangular prism with a 1:3 diameter to height ratio as recommended by the International Society for Rock Mechanics [45], and the end faces of the samples were polished to ensure that the plane error at both ends of the sample was less than 0.02 mm.

### 2.2. Experimental Facility

**Figure 2:**
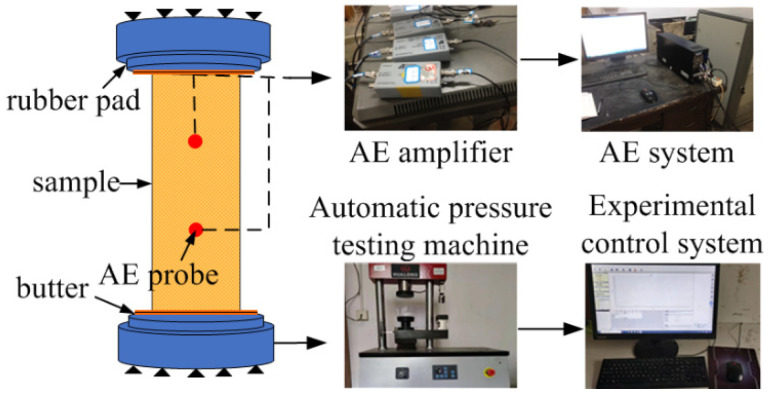
Schematic diagram of the experimental facility.

The automatic pressure testing machine developed by Shanghai Hualong Testing Instrument Company (Shanghai, China) was adopted in this test. The testing machine adopts computer automatic control; using Panasonic servo amplifier and servo motor drive ball screw loading, it can set the loading rate according to the corresponding international standard and meet the standard loading rate control indexes. The experimental facilities are shown in Figure 2. During the experiment, the testing machine selected the loading mode of force control, and the loading rate was kept at 0.1 kN/s, 0.3 kN/s, 0.5 kN/s, 0.7 kN/s, and 0.9 kN/s.

**Figure 3:**
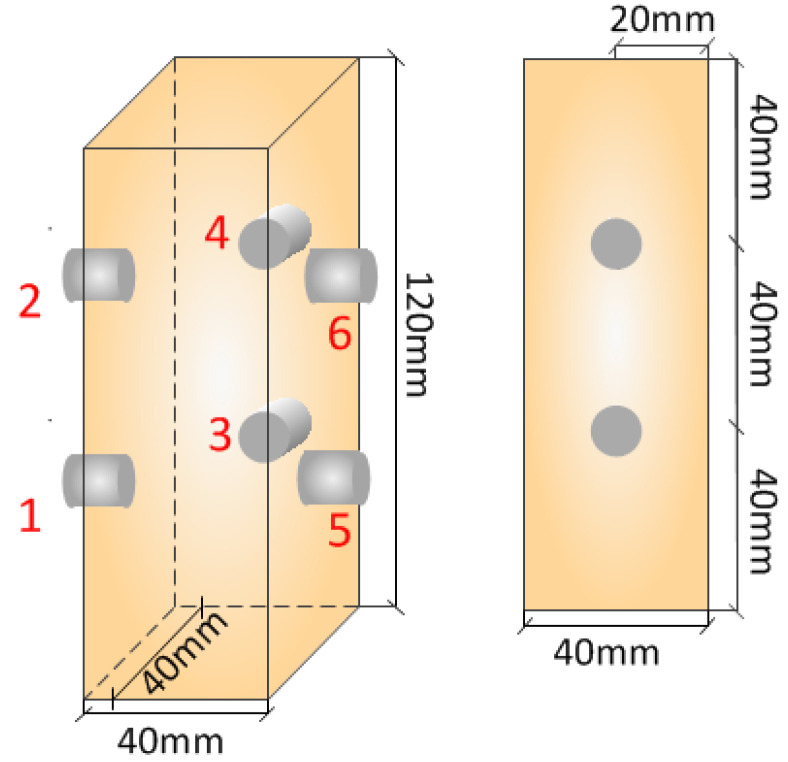
The layout of acoustic emission monitoring points.

An AE system produced by American Physical Acoustics Corporation was employed to collect the AE signals. Six AE sensors were used and numbered 1, 2, 3, 4, 5 6, the AE sensors layout is shown in Figure 3. To improve the contact effect between the AE sensor and yellow sandstone specimen as well as the reception efficiency of AE signals, the specimen surface was smeared with adhesive, the threshold of the AE amplifier was set to 40 dB, and the sampling frequency was set to 2.5 MHz. During the experiment, the loading system and acoustic emission detection were started at the same time, and all experiments were carried out at room temperature, 25 ± 0.5 °C.

## 3. Mechanical Properties and Acoustic Emission Responses

### 3.1. Mechanical Properties

**Figure 4:**
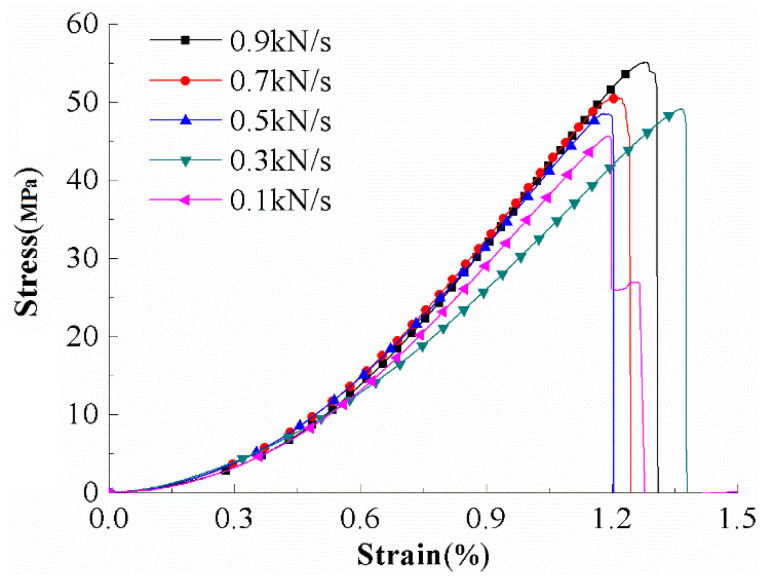
Stress-strain curves of yellow sandstone under different loading rates.

The uniaxial compression stress-strain curves of a group of typical yellow sandstone samples under different loading rates are shown in Figure 4; the stress-strain curves of yellow sandstone under different loading rates are the same. The stress-strain curve can be divided into the initial compaction stage, elastic deformation stage, plastic deformation stage, and post-peak failure stage. In the loading rate range of 0.1–0.9 kN/s, the rock stress-strain curves all show obvious brittleness, and there is no residual stress after the peak.

**Figure 5:**
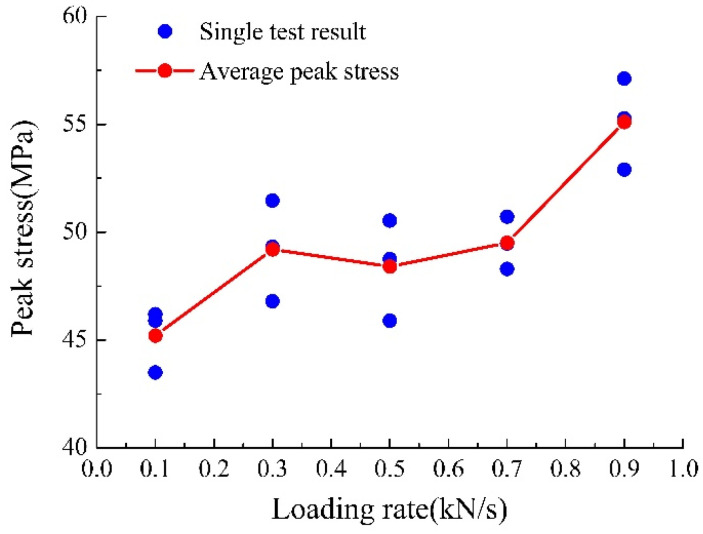
Yellow sandstone’s peak strength under different loading rates.

Yellow sandstone’s peak strength under different loading rates is shown in Figure 5. The experimental results show that peak strength increases with an increase in the loading rate. The uniaxial compressive strength increased from 45 MPa to 58 MPa when the loading rate increased from 0.1 kN/s to 0.9 kN/s; the growth rate was approximately 29%. The increase in peak intensity resembles a hyperbolic function; the increasing trend is large in the 0.1–0.3 kN/s and 0.7–0.9 kN/s ranges, and stable in the 0.3–0.7 kN/s range.

**Figure 6:**
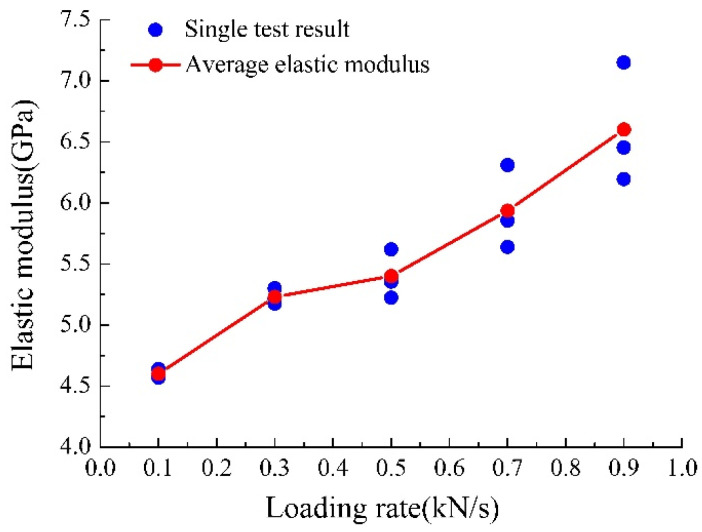
Yellow sandstone’s elasticity modulus under different loading rates.

The elastic modulus of yellow sandstone under different loading rates is shown in Figure 6. When the loading rate increases from 0.1 kN/s to 0.9 kN/s, the elastic modulus increases from 4.6 GPa to 6.6 GPa, the growth rate is approximately 43%, and its change trend is the same as that of the peak strength. Notably, as the loading rate increased, the elastic modulus differences in the same group increased significantly. The reason for this is that an increased loading rate prevents the rock from compacting the bone granules completely; the higher the loading rate, the smaller the compaction density, resulting in a more complex and variable compaction degree. Macroscopically, with an increase in the loading rate, the elastic modulus has greater dispersion.

### 3.2. Acoustic Emission Responses

AE is a rock energy release phenomenon which reflects the rock damage evolution process to a certain extent [46]. According to its definition, the AE parameter can be divided into process parameters and state parameters [47]. The process parameter reflects the AE state during the entire compression process, and is represented by the cumulative energy of AE. The state parameter reflects the AE state at a specific moment, and is represented by the AE ringing count.

**Figure 7:**
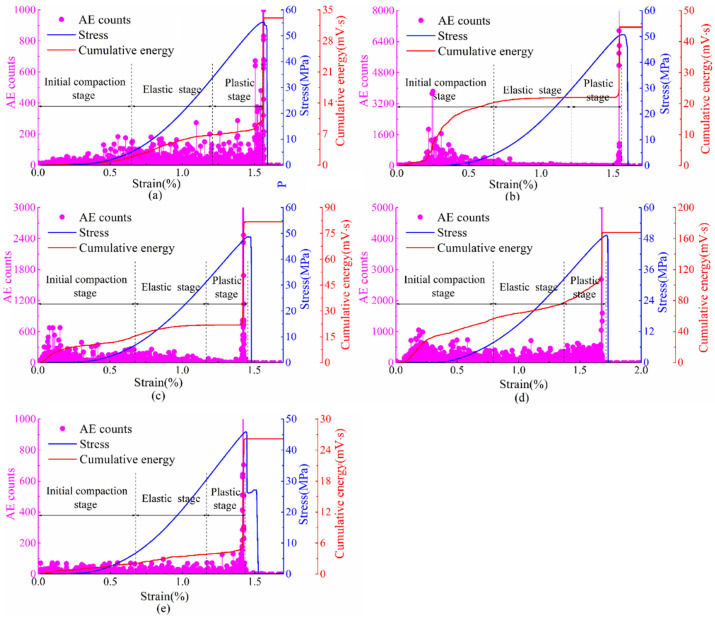
Stress-strain curves, AE ringing count, and cumulative energy diagrams of yellow sandstone under different loading rates: (**a**) 0.9 kN/s; (**b**) 0.7 kN/s; (**c**) 0.5 kN/s; (**d**) 0.3 kN/s; and (**e**) 0.1 kN/s.

The AE ringing count and cumulative energy of yellow sandstone under different loading rates are shown in Figure 7. Corresponding to the stress–strain curve, AE characteristics increase regularly with stress, which can reflect the deformation process of yellow sandstone samples. As the loading increases, AE counts and AE cumulative energy tend to rise and reach the maximum near the peak stress. In the initial compaction stage, more AE counts are generated in a small loading, and the accumulated AE energy increases briefly and rapidly, but it is not significant when the unloading rate is 0.9 kN/s and 0.1 kN/s. Compared to the initial compaction stage, AE counts in the elastic stage do not increase significantly, and accumulated AE energy has a reduced/low growth rate. In the plastic stage, due to the increasing load, new microcracks inside the rock begin to appear, and the continuous expansion and extension of microcracks eventually lead to the appearance of macrocracks and rock damage. During the plastic stage, the AE is more active, showing a rapid increase in accumulated energy and a high ringing count. In the post-failure stage, as the rock completely loses its bearing capacity, the AE activity disappears, showing that the accumulated energy stops growing and the ringing count drops to zero. The AE activity under different loading rates has similar tendencies, which accords with the evolution law of the rock damage process. The results indicate that AE activity can well describe the deformation and failure process of yellow sandstone under different loading rates.

## 4. Construction of Rock Damage Model

### 4.1. Acoustic Emission Damage Model

Under uniaxial compression, the formation and expansion of microcracks inside a rock will release the energy stored in the compression process in the form of waves. In this case, there is an inevitable relationship between rock AE and rock damage evolution. The damage variable D is defined as the ratio of the micro defect section area $A_{d}$ to the initial non-damaged section area A [48]:

(1) $$D=\frac{A_{d}}{A}$$

If the rock is regarded as the state without initial damage, the total number of cumulative acoustic emission events in the entire rock damage region A is $N_{m}$, then the AE rate at the failure of the unit element is:

(2) $$n_{v}=\frac{N_{m}}{A}$$

When the damage section area reaches $A_{d}$, the cumulative AE number is:

(3) $$N=n_{v}A_{d}=N_{m}\frac{A_{d}}{A}$$

According to Equation (3), the damage variable expression can be represented by the cumulative AE number. N is the number of current cumulative AE events.

(4) $$D=\frac{N}{N_{m}}$$

Lemaitre [49] proposed the concept of effective stress and introduced the damage variable (4) into the effective stress relation (5) to define the AE damage model (6):

(5) σ=Eε(1−D)

(6) $$\sigma =E\varepsilon \left(1-D\right)=E\varepsilon \left(1-\frac{N}{N_{m}}\right)$$

where E is the tangent elastic modulus of rock, and ε is rock strain.

### 4.2. Weibull Strength Theory Damage Model

Weibull strength theory holds that the microelement strength of rock is not uniform, and that rock microelement strength obeys the Weibull function distribution law [50]. When combined with the Ducker Prager ideal elastic-plastic criterion, the damage variable expression based on the Weibull strength distribution can be obtained as follows:

(7) $$D=1-\exp\left[-\frac{1}{m}{\left(\frac{\varepsilon }{\varepsilon_{d}}\right)}^{m}\right]$$

(8) $$m=\frac{1}{\ln\left(E\varepsilon_{d}\right)-\ln\sigma_{\max}}$$

where m is the shape parameter related to rock uniformity; $\varepsilon_{d}$ is the average peak strain; and $\sigma_{\max}$ is the maximum stress value.

The Weibull strength theoretical damage model is obtained by putting the damage variable (7) into the effective stress Formula (5).

(9) $$\sigma =E\varepsilon \left(1-D\right)=E\varepsilon \exp\left[-\frac{1}{m}{\left(\frac{\varepsilon }{\varepsilon_{d}}\right)}^{m}\right]$$

### 4.3. Initial Damage Constitutive Model

**Figure 8:**
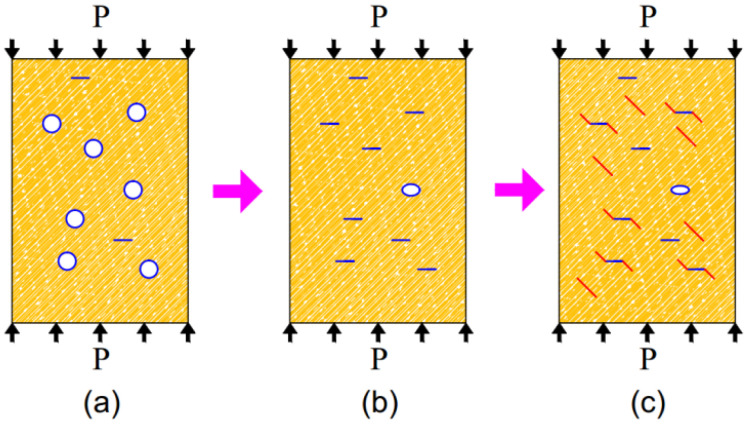
Schematic diagram of rock internal microcrack evolution: (**a**) initial microcracks; (**b**) the compacted initial microcracks; (**c**) new generated microcracks.

Zhang et al. [51], Li et al. [52], Cao et al. [53], and Zhang et al. [54] all regarded rock as skeleton and void, and established the damage model of the rock considering the initial compaction, which is well reflected in the entire process of rock deformation and failure. It shows that the mechanical behavior of brittle rock is inseparable from the rock’s initial microcrack distribution. In a uniaxial compression experiment, the initial microcracks of the rock before compression are shown in the blue circles in Figure 8a. During the initial compaction of the rock, a large number of original microcracks are compressed, which is essentially the self-healing behavior of the rock’s internal structure; the rock’s ability to resist deformation is strengthened, and initial microcrack closure is shown in Figure 8b. As the pressure *P* increases, the rock is gradually broken down by the development of an additional crack. This is a process of compression cracking to produce additional cracks. The additional cracks are caused by rock failure and weaken the rock’s ability to resist deformation, which is represented by the red line in Figure 8c.

**Figure 9:**
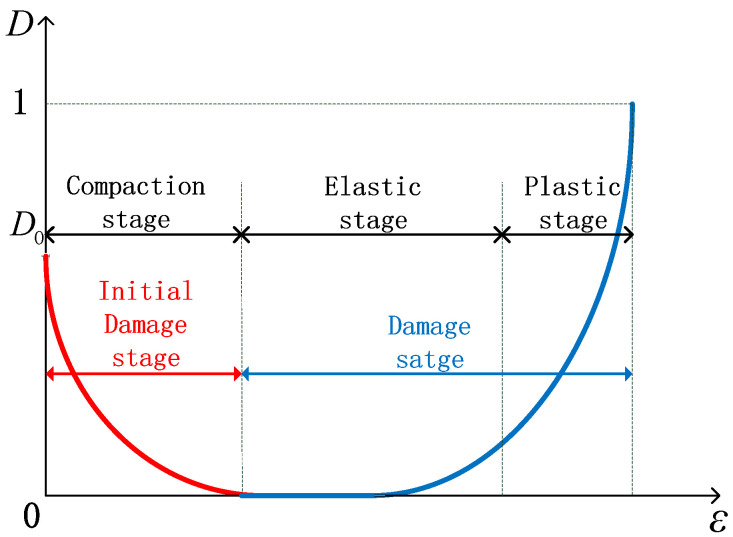
Damage variables vary with strain diagram.

Relative to the rock’s elastic compaction stage, the initial microcracks can be regarded as the initial damage. During uniaxial compression, the minimum number of microcracks stage can be regarded as the starting point of the elastic stage, so the non-damaged state of the rock D = 0 can be defined at the starting point of the elastic stage of rock, as shown in Figure 8b. Remarkably, at this point, there are still microcracks in the rock, which remains different from the completely dense rock. The entire rock damage process is considered to be from the initial damage value $D_{0}$ to 0, and then to 1. The assumed damage variable is shown in Figure 9. According to the skeleton material deformation analysis method in reference [51], the damage variable of defined non-destructive rock relative to dense rock can be calculated as shown in Equation (10), where $E_{s}$ represents the elastic modulus of tight rock.

(10) $$D\prime =1-\frac{E}{E_{s}}$$

Yang et al. [55] introduced the seismological theory, proposed the AE multi-parameters index, and carried out uniaxial compression tests on different sandstone samples (i.e., coarse sandstone, medium sandstone, fine sandstone, and siltstone) with a 0.03 mm/s strain rate. In his studies, the AE damage area experienced a decline, low-value fluctuation, increase, and sustained high-value stage. The AE damage area and damage variable are similar in definition; the change process is consistent with the assumed damage variable, which confirms the rationality of the hypothesis to some extent.

**Figure 10:**
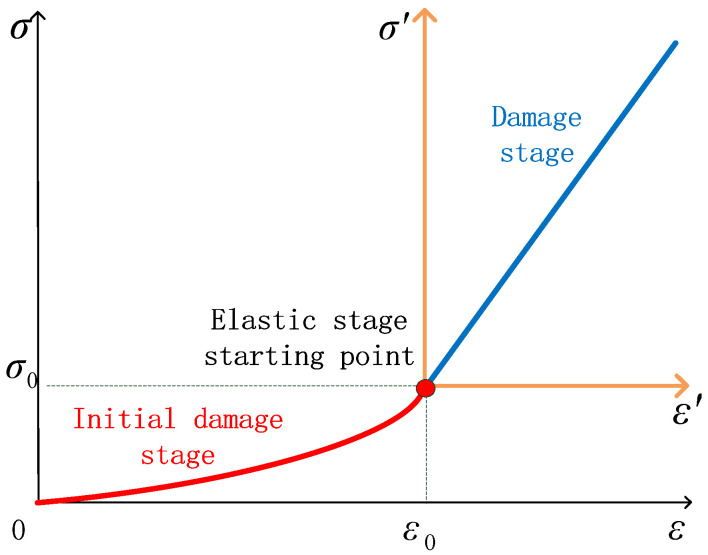
Diagram of axes change.

Under this assumption, using $\varepsilon -\varepsilon_{0}$ to replace ε, the coordinate axis σ−ε transforms into $\sigma^{\prime }-\varepsilon^{\prime }$, as shown in Figure 10. From the stress-strain curve through points (0,0) and ($\varepsilon_{0}$,$\sigma_{0}$) in the coordinate system $\sigma^{\prime }-\varepsilon^{\prime }$, substitute this into the effective stress formula (5); then, the initial damage expression (11) of the initial microcrack state of the rock can be obtained, where $\varepsilon_{0}$,$\sigma_{0}$ represent the corresponding strain and stress values at the starting point of the elastic stage, respectively.

(11) $$D_{0}=1-\frac{\sigma_{0}}{E\varepsilon_{0}}$$

Using Equation (11), the initial damage $D_{0}$, under different loading rates, is shown in Table 1.

Based on the AE count damage model, the cumulative ringing count represents the AE count to reflect the change in D, and the expression of the damage variable D can be obtained:

(12) $$D=\{\begin{array}{cc} \left(1-\frac{N}{N_{m_{1}}}\right)D_{0} & \varepsilon \leq \varepsilon_{0}\\ \frac{N-N_{m_{1}}}{N_{m_{2}}} & \varepsilon >\varepsilon_{0} \end{array}$$

where $N_{m_{1}}$ represents the maximum cumulative AE count in the initial compaction stage; $N_{m_{2}}$ is the maximum cumulative AE count excluding the initial compaction stage; $N_{m_{2}}=N_{m}-N_{m_{1}}$.

**Figure 11:**
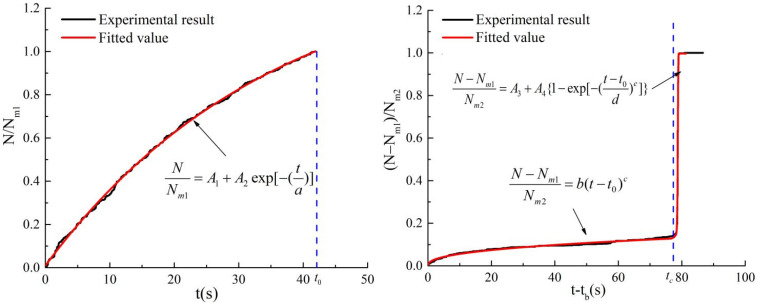
Fitting AE count function diagram.

The results of fitting AE count data are shown in Figure 11, and the fitted parameters under different loading rates are shown in Table 2. All fitting correlation coefficients are over 0.92, which verifies AE counts under different loading rates can be well described. The fitting function is expressed in Equations (13)–(15).

(13) $$\begin{array}{cc} \frac{N}{N_{m_{1}}}=A_{1}+A_{2}\exp\left(-\frac{t}{a}\right) & t\leq t_{0} \end{array}$$

(14) $$\begin{array}{cc} \frac{N-N_{m_{1}}}{N_{m_{2}}}=b{\left(t-t_{0}\right)}^{c} & t_{0}<t\leq t_{c} \end{array}$$

(15) $$\begin{array}{cc} \frac{N-N_{m_{1}}}{N_{m_{2}}}=A_{3}+A_{4}\left\{1-\exp\left[{\left(-\frac{t-t_{0}}{d}\right)}^{e}\right]\right\} & t>t_{c} \end{array}$$

To ensure the continuity of stress, the effective stress formula is modified as follows:

(16) $$\sigma =\{\begin{array}{cc} E\left(\varepsilon -\varepsilon_{0}\right)\left(1-D\right)+\sigma_{0} & \varepsilon \leq \varepsilon_{0}\\ {}[E\left(\varepsilon -\varepsilon_{0}\right)+\sigma_{0}]\left(1-D\right) & \varepsilon >\varepsilon_{0} \end{array}$$

As the loading rate is constant, there exists the relation of *σ* and *t* as shown in Equation (17), where *S* is the sample area and *k* is the loading rate. Combined with Equations (12)–(17), the initial damage constitutive model can be obtained as Equation (18).

(17) t=σS/k

(18) $$\sigma =\left\{ \begin{array}{ll} E\left(\varepsilon -\varepsilon_{0}\right)\left[A_{1}+A_{2}\exp\left(-\frac{\sigma S}{ak}\right)\right]D_{0}+\sigma_{0}, & \varepsilon \leq \varepsilon_{0}\\ {}[E\left(\varepsilon -\varepsilon_{0}\right)+\sigma_{0}]\left\{1-b{[\frac{S}{k}\left(\sigma -\sigma_{0}\right)]}^{c}\right\}, & \varepsilon_{0}<\varepsilon \leq \varepsilon_{b}\\ \left[E\left(\varepsilon -\varepsilon_{0}\right)+\sigma_{0}\right]\left\{1-A_{3}-A_{4}\left\{1-\exp\left[-{\left(S\frac{\sigma -\sigma_{0}}{kd}\right)}^{e}\right]\right\}\right\}, & \varepsilon >\varepsilon_{b} \end{array}\right.$$

### 4.4. Verification of the Initial Damage Constitutive Model

**Figure 12:**
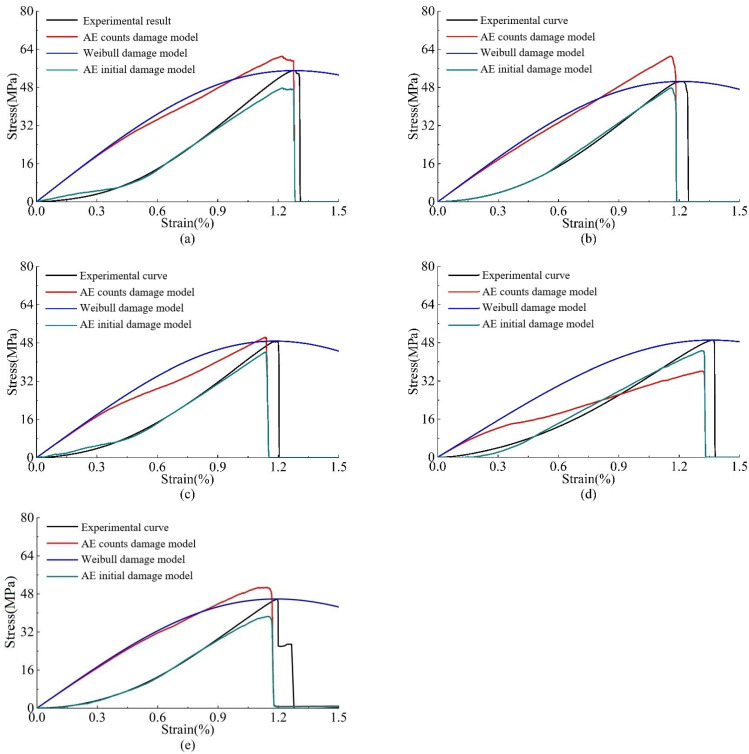
Experimental stress-strain curves of AE damage model, Weibull strength statistical damage model, and AE initial damage model under different loading rates: (**a**) 0.9 kN/s; (**b**) 0.7 kN/s; (**c**) 0.5 kN/s; (**d**) 0.3 kN/s; and (**e**) 0.1 kN/s.

The AE damage model, the Weibull strength statistical damage model, and the initial damage model are compared to the experimental results at different loading rates, as shown in Figure 12. The AE damage model and the Weibull strength statistical damage model reflect the elasticity compression stage well. The AE damage model is better than the Weibull strength statistical damage model when reflecting the stress-strain curve after the peak; however, there are great differences between the two curves and the measured curves in the initial compaction stage. The initial damage constitutive model in this paper overcomes this defect and is closer to the measured results in the entire stress-strain stage.

## 5. Conclusions

This study conducted a series of mechanical tests for yellow sandstone subjected to monotonous loading under different loading rates, and analyzed the effects of the loading rate on the mechanical behaviors and AE characteristics of yellow sandstone. Finally, it established an initial damage constitutive model based on the AE count, and compared this with other methods. The following conclusions can be drawn:

1. The mechanical properties of yellow sandstone are sensitive to the loading rate. The uniaxial compression strength and elastic modulus are highly dependent on the loading rate and increased by 21.7% and 43.2%, respectively.

2. The established initial damage constitutive model considered the initial compaction stage of yellow sandstone subjected to monotonous compression with different loading rates, which can better describe the damage evolution process and the total stress-strain curve of yellow sandstone.

Based on the AE results of yellow sandstone under uniaxial compression, a damage constitutive model was established, which considered the rock’s initial damage. Verification results show that the established initial damage constitutive model can reflect rock damage more accurately. However, this model does not study rock damage from the perspective of petrography. In future studies we will further study rock damage from the perspective of petrography.

## Figures and Tables

**Table 1:** Initial damage $D_{0}$ under different loading rates.

Loading Rate (kN/s)	0.1	0.3	0.5	0.7	0.9
$D_{0}$	0.591	0.626	0.572	0.637	0.624

**Table 2:** Fitted parameters under different loading rates.

Loading Rates (kN/s)	Fitted Parameters								
	$A_{1}$	$A_{2}$	a	b	c	$A_{3}$	$A_{4}$	d	e
0.1	1.463	−1.443	47.466	0.024	0.680	0.130	0.874	96.330	436.378
0.3	1.430	−1.458	38.246	0.017	0.330	0.073	0.838	63.596	378.388
0.5	1.446	−1.436	35.746	0.014	0.340	0.035	0.964	88.018	160.209
0.7	1.097	−1.100	6.004	0.021	0.401	0.142	0.855	78.784	681.369
0.9	1.480	−1.401	21.925	0.002	1.077	0.147	0.848	46.706	203.098

## Data Availability

Data are contained within the article.
